# Data Mining for Wearable Sensors in Health Monitoring Systems: A Review of Recent Trends and Challenges

**DOI:** 10.3390/s131217472

**Published:** 2013-12-17

**Authors:** Hadi Banaee, Mobyen Uddin Ahmed, Amy Loutfi

**Affiliations:** Center for Applied Autonomous Sensor Systems, Örebro University, SE-70182 Örebro, Sweden; E-Mails: mobyen.ahmed@oru.se (M.U.A.); amy.loutfi@oru.se (A.L.)

**Keywords:** data mining, wearable sensors, healthcare, physiological sensors, health monitoring system, machine learning technique, vital signs, medical informatics

## Abstract

The past few years have witnessed an increase in the development of wearable sensors for health monitoring systems. This increase has been due to several factors such as development in sensor technology as well as directed efforts on political and stakeholder levels to promote projects which address the need for providing new methods for care given increasing challenges with an aging population. An important aspect of study in such system is how the data is treated and processed. This paper provides a recent review of the latest methods and algorithms used to analyze data from wearable sensors used for physiological monitoring of vital signs in healthcare services. In particular, the paper outlines the more common data mining tasks that have been applied such as anomaly detection, prediction and decision making when considering in particular continuous time series measurements. Moreover, the paper further details the suitability of particular data mining and machine learning methods used to process the physiological data and provides an overview of the properties of the data sets used in experimental validation. Finally, based on this literature review, a number of key challenges have been outlined for data mining methods in health monitoring systems.

## 1. Introduction

With the increase of healthcare services in non-clinical environments using vital signs provided by wearable sensors, the need to mine and process the physiological measurements is growing significantly.

Moreover, recent advances in data mining for health monitoring systems have led to provide proactive information [1]. In the literature much attention has been given to sensor development and architectures for wearable sensors [2–5]. Today, there are several reviews on the topic which provide an overview of the current state of the art [6–9]. More specifically these reviews contribute with a general and global overview of wearable sensors and their relevance to biomedicine, medical informatics, and ambient assisted living (AAL) [10–12]. However, as the field progresses and more works consider deployment in real settings, data mining techniques that consider the specific challenges which emerge from data coming from wearable sensors is of ever increasing importance. In health monitoring systems focus has been recently shifting from that of obtaining data to one of developing intelligent algorithms to perform a variety of the tasks [12]. Such tasks not only include traditional pattern recognition and anomaly detection but also must consider decision based systems which can handle context awareness, and subject specific models and personalization. These latter challenges are particularly important if health monitoring services are to be designed that can address the growing market needs and opportunities in pervasive sensing such as distributed health monitoring and long-term prevention. This paper attempts to clarify how certain data mining methods have been applied in the literature. It also attempts to reveal trends in the selection of the data processing methods based on the requirements of the monitoring system. For this reason, the paper focuses on reviewing the data mining and pattern recognition methods used in the literature for applications involving wearable sensing technologies. Focus is put on the algorithms and data sets that have been used in order to provide an overview of the algorithm's capabilities and shortcomings.

In this review paper we provided a collection of relevant works in this field to cover recently applied data processing methods on wearable sensor. As the considered area consists both within the computer science and health or medical science, the range of possible terminologies to consider were wide. Therefore, some global instances of the investigated keywords in this study are: “wearable sensors, health monitoring, Sensor data mining, physiological time series, healthcare, health informatics, healthy living and wellbeing, physiological sensors, health monitoring, machine learning technique, vital signs monitoring, biomedical signal processing, and pattern recognition”.

As the literature in this field is vast, we also have limited the scope of this paper to include wearable sensors that measure health parameters such as vital signs for disease management and prevention health monitoring systems. Specifically we concentrated the review on the following vital sign parameters: electrocardiogram (ECG), oxygen saturation (SpO_2_), heart rate (HR), Photoplethysmography (PPG), blood glucose (BG), respiratory rate (RR), and blood pressure (BP). Also this survey covered the other synonym terms and specific disease or problems related to the mentioned health parameters. Also for data analysis part, we combined the mentioned keywords with the particular terms in data mining, machine learning, pattern recognition and decision support system fields in order to gather the articles which contain data processing phase. It should be noted that the activity monitoring via accelerometers such as activity recognition, gait research and rehabilitation is outside of the scope of this review. The following studies [13–16] have provided the recent reviews on the topic of human activity monitoring. Finally, for the sake of investigating the recent trends of research in mentioned area and also eliciting the potential challenges, the period of the collected papers are filtered to the last four years. So, the selected papers as the representatives of the performed research are limited to the more than eighty papers.

The paper is organized as follows. In Section 2, we first begin with a brief summary of review works which are relevant to this survey paper. The subsequent sections then delimit the recent related literature first by which data mining tasks are done with wearable sensor data (Section 3), then by the specific algorithms used to achieve these tasks (Section 4). Then, a description of the type of data sets which are collected using wearable sensors is given in Section 5. Finally, the paper concludes with a discussion of future challenges in Section 6 and a conclusion in Section 7.

## 2. Related Review Papers

Several comprehensive reviews about the subject of health monitoring with wearable sensors have been previously presented in the literature. Many such reviews focus on giving a global overview of the topic. Studies on health monitoring systems include wearable, mobile and remote systems [10]. Most notably, are works such as [10,17] which focus on the needs to have wearable sensors and overcoming important bottlenecks for the use of wearable sensors such as the clinical acceptability and interoperability in health records. According to [17,18], smart wearable systems supports complex healthcare applications and enable low-cost wearable, non-invasive alternatives for continuous 24-h monitoring in Bioinformatics, imaging informatics, clinical informatics, and public health informatics. However, in real time analysis of sensor data while considering mobile health monitoring systems, the time of data analysis and resources for data processing is important as it is presented in [10]. Similar works have been done by [12,19] where the authors also provide a useful mapping between specific diseases and which vital sign parameters can be measured are relevant for the disease. Such diseases and ailments include: cardiovascular diseases, respiratory diseases, diabetes mellitus, renal diseases, posture and motion control, rehabilitation, Parkinson's disease, stress, neurological disorders, Alzheimer's disease and dementia [10]. More sensor data (except the mentioned vital signs) have been considered in the literature such as EEG and body temperature [17]. Nevertheless, integration and interpretation of the sensor signals while considering the state of a patient is still challenging [19]. Similarly, according to [12], subject-specific models in data mining and context aware in data analysis are still unsolved.

Considering the data mining reviews for healthcare and sensors, today, most of them are related to general studies for healthcare *i.e.*, well known problems in healthcare with simple and routine data mining approaches [19]. Recently, Sow [1] categorized the main challenges of sensor data mining in five following stages: acquisition, preprocessing, transformation, modelling and evaluation. The authors in [18,20] found the data mining algorithms mainly in two categories (1) descriptive or unsupervised learning (*i.e.*, clustering, association, summarisation) and (2) predictive or supervised learning (*i.e.*, classification, regression). However, they are lacking deeper insight into the suitability of the algorithms for handling the special characteristics of the sensor data in health monitoring systems.

## 3. Data Mining Tasks for Wearable Sensors

**Figure 1.**
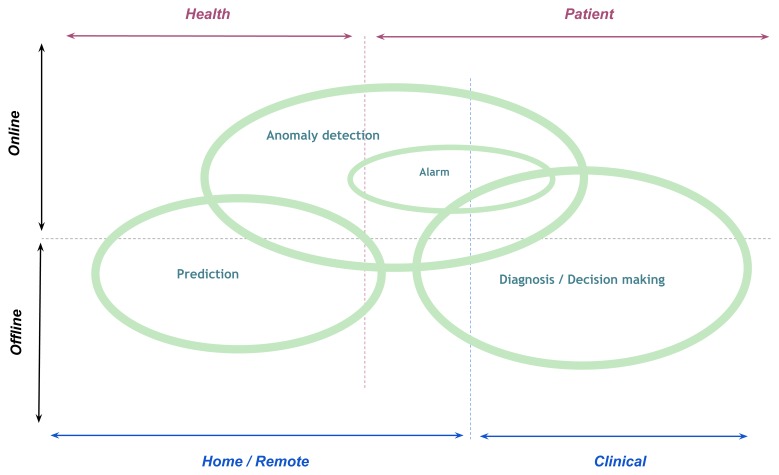
A schematic overview of the position of the main data mining tasks (anomaly detection, prediction, and diagnosis/decision making) in relation to the different aspects of wearable sensing in the health monitoring systems.

Recently, the research area of health monitoring systems has shifted from simple reasoning of wearable sensor readings (like calculating the sleep hours or the number of steps per day) to the higher level of data processing in order to give much more information that is valuable to the end users. Therefore, healthcare services have been focusing on deeper data mining tasks to have deeper knowledge representation. Based on the selected literature, three types of data mining tasks are predominant. These three tasks are: prediction, anomaly detection which may include the subtask of raising alarms, and diagnosis where a decision making process is made to often categorize the data into different groups depending on the diseases. Each of these tasks is further described in this section. Figure 1 provides a depiction of each task in relation to three dimensions. The first dimension involves the setting in which the monitoring occurs. Based on the literature, most monitoring applications which consider home settings or remote monitoring deal predominantly with prediction and anomaly detection whereas the applications in clinical settings are typically focused on diagnosis [10,21]. This fact is easily explained by the growing desire to have a more preventative approach (prediction) via wearable sensors and to consider the possibility to facilitate independent living in home environments by increasing the sense of security (alarm). Similarly, in clinical settings much more information is available in order to provide diagnosis and assist in decision making [18]. A second dimension in the Figure shows the main data mining tasks in wearable sensors with respect to the type of subjects used. For patients with known medical records, both diagnosis and specifically the possibility to raise alarms are key tasks. For health monitoring which typically include healthy individuals who want to ensure the maintenance of good health, prediction and anomaly detection are used in the literature [22]. The final dimension depicted in the Figure considers the three main data mining tasks in relation to how the data is processed. For all three tasks data has been addressed both in an online and offline manner, with more alarm related tasks being naturally used in the context of online and continuous monitoring [1].

**Figure 2.**
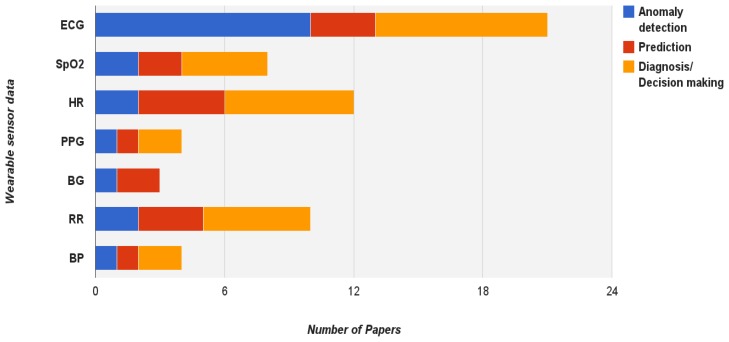
The outline of the distribution of three data mining tasks in relation to the vital signs measured by wearable sensors considering in this review.

Figure 2 provides an outline of the three most used data mining tasks in relation to the vital signs that can be measured by wearable sensors considered in this review. ECG, which provides mostly the rich data, is predominantly used for all tasks in comparison to the other types of sensors. Equal emphasis is placed on anomaly detection and diagnosis and less attention put on prediction. In general, this applies to the majority of the vital sign parameters in the Figure. However, for health parameters such as heart rate and blood glucose, the percentage of the tasks dedicated to predication is higher. This is most likely due to the fact that these parameters are associated with monitoring of specific diseases e.g., diabetes whereby prediction is an important component [23]. This section further details the definition of each task depicted in Figures 1 and 2 and provides key related works for each task.

### 3.1. Anomaly Detection

Anomaly detection is the task of identifying unusual patterns which do not conform to the expected behavior of the data [24]. The retrieved abnormal patterns in physiological data are significantly valuable in the medical domain. Detected unusual patterns in health parameters, especially for home monitoring systems, enables the clinicians to make accurate decisions in short time [25]. Anomaly detection techniques are often developed based on a classification methods to distinguish the data set into normal class and outliers [26]. For example, support vector machines [27], Markov models [28] and Wavelet analysis [29] are used in health monitoring systems for anomaly detection.

According to our review, most of the papers using the anomaly detection approach usually deal with short term [30] and multivariate data sets [31] in order to characterize the entire the data to find discords. Some of the papers considered finding irregular patterns in vital signs time series such as abnormal episodes in ECG pulses [31,32], SpO_2_ signal [33] and blood glucose level [28], which mostly discover unusual temporal patterns in continuous data. Few of the papers have used domain knowledge and predefined information to detect anomalies for decision making such as anomaly detection in sleep episodes [34,35], and finding hazardous stress levels [36]. In online health monitoring systems, raising alarm as soon as detecting any anomaly in vital signs will be triggered to have instant reaction and such alarm system are usually designed for monitoring patients in clinical units [33]. However, anomaly detection task could use offline techniques in order to detect abnormal reading for each individual based on the historical measurements for the person [28], since the definition of the abnormal patterns can be different for each subject.

### 3.2. Prediction

Prediction is an approach that is widely used in data mining field that helps to identify events which have not yet occurred. This approach is getting more and more interest for the healthcare providers in the medical domain since it helps to prevent further chronic problems [18] and could lead to a decision about prognosis [37]. The role of the predictive data mining considering wearable sensors is nontrivial due to requirement of modeling sequential patterns acquired from vital signs. This approach is also known as supervised learning models [20] where it includes feature extraction, training and testing steps while performing the prediction of the data behavior. As the common examples of the predictive models, authors in [38,39] presented a method which predicts the further stress levels of a subject. A similar example of using predictive models in healthcare are: blood glucose level prediction [23], mortality prediction by clustering electronic health data [40], and a predictive decision making system for dialysis patients [41]. For the sake of the unexpected situations and conditions in environmental health monitoring (e.g., home), the difficulty of using predictive models is higher than controlled positions such as clinical units. However, there are several new prediction works which have used experimental wearable sensor data to perform non clinical health monitoring [26,42].

### 3.3. Diagnosis/Decision Making

Decision making in diagnosis is one of the main tasks of clinical monitoring systems which is often based on retrieved knowledge using vital signs, and also other information such as electronic health records and metadata [43]. The diagnosis/decision making is related to the anomaly detection in data mining in order to extract useful information of sensor data such as events, outliers, and alarms which are meaningful for decision [8]. However, a distinction is that diagnosis systems are not necessarily using abnormal patterns in vital signs to make decisions. Moreover, the complication of the conditions, especially about patient's situations, needs more robust and global information rather than sensor's abnormal patterns alone [43]. Hence, in this paper diagnosis/decision making is considered as a task in processing physiological data. Some examples of recent works in this area involve estimating the severity of health episodes of patients suffering chronic disease [44–46], sleep issues such as polysomnography and apnea [47,48], estimation and classification of health conditions [49,50], and emotion recognition [51]. Most of these researches have used online databases with annotated episodes in order to have sufficient and trustable real-world disease labels to evaluate the decision making process. Considering the complexity of the data to infer diagnosis, some researchers frequently used classification methods on short term clinical data such as Neural Network (NN) [52] and decision trees [51].

### 3.4. Other Data Mining Tasks for Wearable Sensors

The main role of data mining in healthcare monitoring systems is retrieving information (*i.e.*, anomaly detection, prediction and diagnosis decision making), and there are several tasks considering wearable sensors that data mining methods are able to carry out. According to [1,19], most healthcare systems are dealing with the following issues: (1) data acquisition using the adequate sensor set; (2) transmission of data from subject to clinician; (3) integration of data with other descriptive data; and (4) data storage. Considering these issues leads to investigate some data mining tasks such as data cleaning, noise removing, data filtering and compressing as a part of any physiological data monitoring frameworks. In order to conduct these problems, several data mining techniques are applied such as wavelet analysis for artifact reduction [53] and data compression [54], rule-based methods for data summarizing and transmitting [55,56], and Gaussian process for secure authentication [57]. These kinds of tasks appeared due to this fact that of real-world health monitoring systems are usually dealing with unlabeled and continuous data [17].

## 4. Data Mining Approach

**Figure 3.**
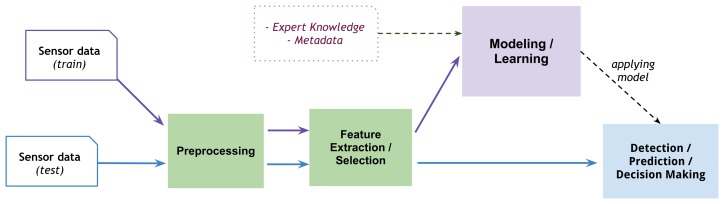
A generic architecture of the main data mining approach for wearable sensor data.

In health monitoring systems, the role of data analysis is to extract information from the low level sensor data and bridge them to the high level knowledge representation. For this reason recent health monitoring systems have given more attention to the data processing phase in order to catch more valuable information based on the expert user requirements. Data mining techniques that have been applied to wearable sensor data in health monitoring systems have varied and it is also not uncommon that several techniques are used within the same architecture. In this section we outline the most common approaches used with wearable sensor data to provide valuable information such as mentioned tasks in Section 2. Regardless of the data mining technique used, the most standard and widely used approach to mining information from wearable sensors is given in Figure 3.

In the figure, the raw sensor data is typically used as a starting point of the data mining approach. Here, the sensor data is provided for both training data in order to learn the system, make a model of features, as well as testing data for real-world usage designed model and make the result. This data mining approach is suggested as a general flow for both supervised and unsupervised data mining solutions in order to provide any kind of data mining task as result. The main steps of the data mining approach consist (1) data preprocessing; (2) feature extraction and selection; and (3) modelling data learning the input features (considering expert knowledge and metadata) to perform the tasks such as detection, prediction, and decision making.

It should be noted that other parameters on data mining and machine learning methods are important such as expert knowledge, historical data measurements, electronic health records, and stable parameters (e.g., sex, age). This metadata provides contextual analysis and improves the process of knowledge extraction [29,57]. An instance is every healthcare system using HR sensor data needs to investigate the effect of metadata such as age, sex, weight, and medicine in order to have meaningful reasoning to find *i.e.*, basic abnormal heart rates or to personalize the critical pulses based on the mentioned metadata [58,59]. The details of the main steps in the data mining approach are presented below:

### 4.1. Preprocessing

Due to the occurrence of noise, motion artifacts, and sensor errors in any wearable sensor networks, a preprocessing of the raw data is necessary. Preprocessing in the healthcare domain involves (1) filter unusual data to remove artifacts and (2) remove high frequency noise [1,13]. According to the literature, for filtering artifacts, designed modules have usually applied threshold-based methods to filter sensor data [36,60] or used statistical tools to interpolate the missing data points [61]. For example in ECG data, several works have been done to improve the quality of signals for accurate analysis [29,32]. To remove frequency noise, the other methods in frequency domain such as such as power spectral density (PSD) fast Fourier transforms (FFT), and low-pass/high-pass filtering tools are common to remove the fluctuations in sensor signals [51,62]. When the data is gathered from numerous wearable sensors, normalization and synchronization of sensor data is required. The main challenges of the preprocessing phase in healthcare systems are addressed in [1] which includes data formatting, data normalization, and data synchronization. However, there is no tailored work considering these issues in detail for real life scenarios. Since the gathered sensor data is often unreliable and massive, the papers working with large scale and continuous data necessarily employed a preprocessing step [45].

### 4.2. Feature Extraction/Selection

Generally, for mining massive and real world data sets, the abstraction of raw data in any data mining approach is a way to design and build a model in order to retrieve valuable information. The aim of feature extraction is to discover the main characteristics of a data set which are identically representatives of the original data [63]. Especially in wearable sensor data, according to the magnitude and complexity of the raw data, feature extraction provides a meaningful representation of the sensor data which can formulate the relation of raw data with the expected knowledge for decision making [64]. Moreover, reducing the amount of sensor network data is another task in feature extraction and feature selection phases which leads to have an arranged vector of features as an input of data mining techniques like classifier methods [45,60].

Since Wearable sensor data which provide monitoring of vital sign parameters tend to be continuous time series readings, most of the considered features are related to the properties of time series signals [42]. Two main aspects of analyzing signals are time domain and spectral domain [14]. In the time domain, the extracted features usually include basic waveform characters and statistical parameters related to the visible attributes in data stream such as mean, variance, pick counts, *etc.* [36,61]. In physiological data, the time-domain features are common, because the traditional decision making frameworks on vital signs are based on the observable trends in the signal [33]. However, for extra knowledge about the periodic behavior of time series, research in the medical field has given more attention to the features acquired from frequency-domains such as power spectral density, low-pass/high-pass filters, spectral energy, and wavelet coefficients of the signal [29,45]. Table 1 summarises the most appeared features in the literature that extracted from wearable sensor data. As an example, for the works studying ECG signals, although Bsoul *et al.* [65] have proposed dozens of features, the main focus of research is to consider R points (the pick point of each beat), and its properties in ECG pulses such as R-R intervals, pick counts, *etc.* [39,64]. Besides the features from these domains, there are other considered features which such as heuristic and specific field features [61].

Depending on the size of the extracted features from raw data, and the ability of the learning method to handle these data, feature selection is an effective solution in order to select more discriminative features. Feature selection methods usually find a subset of high dimensional data which are more irrelevant and contribute to the performance of the learners [60]. For physiological data, using feature selection techniques leads to reduce the dimensions of input data. Three most popular approaches for dimension reduction in medical domain are PCA, ICA, and LDA [66] which are statistically selecting the subset of the most significant features [45,67]. Other tools for feature selection used in the literature includes threshold-based rules [61], analysis of variance (ANOVA) [51], and Fourier transforms [52].

Although most of the proposed frameworks in the healthcare domain contain feature extraction/selection phases, the main challenge still is to balance between the optimum feature extraction/selection methods and their costs for the system. For instance, it would be costly to use feature selection in real time systems since the modeling techniques can handle the raw extracted features. In these cases, the framework has to deal with unnecessary redundancies which reduce the accuracy of results. The mentioned challenge is directly related to considering (1) selected health parameters in the system and (2) data mining tasks or the target of the health monitoring system. However, its solution is still to be addressed in recent research.

### 4.3. Modeling and Learning Methods

When such sensors are deployed in applications such as home monitoring, a large amount of data can be generated. Further, when multiple sensors are used, this data is multivariate with possible dependencies. This means that in order to make sense of the data, appropriate data processing techniques are essential [1]. In this section we outline the most common algorithms used with wearable sensor data. Each algorithm is given in technical detail with the most representative examples on how the algorithm has been applied in healthcare services. In addition, the usability, efficiency and challenges of each technique in the medical domain are indicated.

#### 4.3.1. Support Vector Machines

A support vector machine (SVM) is one of the main statistical learning methods which is able to classify unseen information by deriving selected features and constructing a high dimensional hyper plane to separate the data points into two classes in order to make a decision model [69]. As the SVM has the ability to handle high dimensional data using minimal training set of features, it is recently very popular for mining physiological data in medical applications.

Common health parameters considered by SVM methods are ECG, HR, and SpO_2_ which are mostly used in the short term and annotated form. Hu *et al.* [62] used SVM to find out arrhythmia in ECG signals. It applied a binary classifier version of SVM to categorize ECG signals into normal and arrhythmia classes. Similarly, [27] developed an SVM method for detecting the arrhythmia and seizure episodes while ECG signals. This research showed that the SVM method with polynomial kernel leads to better result rather than other kernels. Detection of the patient's condition is the topic of [44] which used a one-against-all SVMs method in order to handle multi-label classification. Based on the expert's labels on data episodes (4 levels of the severity), several binary SVMs with different kernels such as RBF, polynomial, and sigmoid are combined to model the input data from multi sensor features. Special usage of SVM classifier is addressed in [32]. This work identified the bad notch in the subjects of chronic gastritis and arthritis with binary classifications of normal and abnormal radial pulses of ECG using the SVM algorithm. The input of the method the reduced power spectral density features of pulse. The performance of the method is evaluated with the sensitivity, specificity, and accuracy of the results.

In general, SVM techniques are often proposed for anomaly detection and decision making tasks in healthcare services. However, SVM is not an appropriate method to integrate domain knowledge in order to use metadata or symbolic knowledge seamlessly with the measurements from the sensors. Moreover, like other classifiers, SVM could not be applied to find the unexpected information from unlabelled data.

#### 4.3.2. Neural Networks

A Neural Network (NN) is an artificial intelligent approach which is widely used for classification and prediction [70]. The NN method models the train data by learning the known classification of the records and comparing with predicted classes of the records in order to modify the network weights for the next iterations of learning. Due to the admissible predictive performance of NN, it is presently the most popular data modeling method used in the medical domain [37]. The ability of the NN is to model highly nonlinear systems such as physiological records where the correlation of the input parameters is not easily detectable [71].

A wide range of the diagnosis and decision making tasks has been done by NN in the medical domain. NN has also been applied for multi-sensor networks in order to handle the sophisticated analysis of multivariate data. The multi-layer perceptron (MLP) neural network has been applied in [72] to estimate the quality of the pulses in PPG. This network puts several individual signal quality metrics as input and then optimizes the number of nodes (2–20) hidden layer in validation iterations. The result performs in two classes of signal quality as output. For evaluation of the work, it employed some common indices such as sensitivity, specificity, and accuracy. Vu *et al.* [49] proposed a framework to recognize Heart Rate Variability (HRV) patterns using ECG and accelerometer sensors. This method used a three layer neural network to incrementally learn the extracted patterns and classify them. Three nodes in the output layer identified three classifications of data for location, activity and heart status. Replicator Neural Network (RNN) [73] is another type of NN which is generally used for anomaly and outlier detection. Recently, Chatterjee *et al.* [23] suggested an RNN to predict blood glucose levels. They designed the network with 11 input variables, one output node as predicted BG level and three hidden layers each with 8 neurons. Online classification of sleep/awake states is another research [52] based on a feedforward neural network on ECG and RR features in the frequency domain. This network is designed in three different architectures (without hidden layer) which differed in the type of input signal. For evaluation, this work used other clinical parameters (e.g., EEG) to monitor and label the collected data. Other examples of the works who has dealt with NN are [45,74,75].

In sum, since the progress of learning in NN would be complex, the method is commonly used for decision making in clinical conditions with large and complicated data sets. But same as SVM it could not handle domain knowledge to enrich the results. Additionally, as the modeling process in NN is a black box progress, NN method needs to justify for each input data. So, NN is not counted as a portable technique to easily apply for diverse data sets.

#### 4.3.3. Decision Trees

To make an accurate discrimination for selected features, the decision tree method is one of the significant learning a technique which provides an efficient representation of rule classification [51]. In this method, the most robust features have been detected for initial splitting the input data by creating a tree-like model. Decision tree is a reliable technique to use in different areas of medical domain in order to make a right decision [76,77]. Nowadays, upon dealing with complex and noisy data, the C4.5 algorithm is used which is estimating the error rate of initial nodes and pruning the tree to make a more efficient sub-tree [41,51].

More attention has been given to decision trees in medical domain when short term data with few numbers of subjects has been used. This method is also suitable for handling multivariate sensors due to construction of independent levels in the decision tree. Frantzidis *et al.* [51] provided a new technique for motion recognition. It used C4.5 decision tree algorithm based on the Mahalanobis distance to divide positive or negative emotions of subject while viewing affective pictures. The tree is designed using the features of biosensor data in two layers of emotion discriminations. The applied classifier used a rule-based method in order to make a proper decision. Prediction of heat stress risk is another classification task [26] which is considered with decision trees. This method used the input parameters such as mean skin temperature, cooling actuation, and ambient temperature and with applying some rules in order to make a C4.5 tree. The output of the tree is a label of danger or safe with 95% accuracy. Another suggested application of decision tree in this field is addressed by Bellos *et al.* [46,64]. In [46] a decision support system is developed to classify the severity of health level using Random Forest (RF) classification as a version of the decision tree. This method used a fusion system to combine the severity of chronic disease for ECG and HR data. For the construction of each tree of the forest, a new subset of the features was picked. For selecting the best tree, the method used threshold-based rules. The accuracy of the system has been checked with some predefined targets. Other applications of decision trees in healthcare services are mentioned in [29,39,41]

Generally, decision tree methods are limited to the space of the constructed features as the inputs of the model. So, finding hidden information out of constricted features would not be recognizable. Furthermore, since the number of features can impact on the efficiency of the method, decision tree models are not usually applied to big and complex physiological data. However, all the mentioned tasks in Section 3 have been addressed in the literature with a decision tree solution, because it is simple and easy to implement.

#### 4.3.4. Gaussian Mixture Models

Gaussian mixture model (GMM) is another statistical model which used for classification and pattern recognition [57]. This method is based on this assumption that the input data are a linear combination of Gaussian distributions. After random initializing some parameters as the first step of the method, the model in GMM is re-estimated based on the raw data and computed the conditional probability density of each target class [45]. Studies using GMM usually deal with annotated medical data in order to assess the performance of the model. Wang *et al.* [57] proposed a GMM method using inter-pulse interval (IPI) signals of ECG in order to make the secure body sensor communications. This tool is based on the fact that the ECG signal behavior is unique for each person and could be used as a signature for authenticating other knowledge (e.g., medication delivery content information). Clifton *et al.* [31] presented a principled approach to increase the ability of personalizing patient monitoring using Gaussian process. This framework used Gaussian process to estimate reliably the distribution of the values of the physiological data. Then it detects anomalous in SpO_2_ channel using the mean function of Gaussian process. The method has been developed to improve removing artifacts and missing data from individual subjects. Another research using GMM is a recent work [45] proposing an automatic detection the coronary artery disease conditions with ECG signals. This method used several classification methods (*i.e.*, SVM, NN, GMM) in order to categorize signal episodes to normal and chronic state. The results of this work showed that GMM classifier provided highest accuracy.

Despite GMM is able to detect unseen information in physiological data, it has been rarely used for prediction tasks. Since the computation time of constructing the models is high, applying GMM in real-time frameworks is not affordable. Moreover, the initialization step in GMM does not necessarily enrich the modeling (as GMM approached assume data as a combination of Gaussian distinction) due to the character of health parameters in real world situations.

#### 4.3.5. Hidden Markov Models

Hidden Markov model (HMM) is a probabilistic model which is convenient for modeling sequential data [78]. Usually, signals are modeled as a Markov chain to compute the probability of each state's occurrence by calculating a histogram of the probabilities of successive states [79]. Using this model the hidden states can be inferred from the other observations in sequence of data [80]. Zhu *et al.* [28] presented an HMM model to detect the abnormal values in measured blood glucose level. In this work, the HMM used the history of normal measurements of each person as a benchmark and then it finds the unusual data in a test set. The state transition diagram of the HMM includes sleep, fasting, and meal states which are connected with the probability of the BG level for each transition. Another work on HMM focused on detecting salient segments in the discovered motifs of wearable sensor time series [81]. The performance of this model was evaluated by ECG and accelerometer sensor data. This work used Markov chain to model the signals with computing the probability of each segment. Segments with the lowest probabilities are counted as salient (only interesting segments).

In general, HMM is used for anomaly detection rather than any other tasks. Additionally, to the best of our knowledge, this method has not been applied to multi health parameters analysis and big data sets. However, based on the abilities of HMM to model the unexpected behaviors of the data, it is applicable to use the modified versions of HMM in more problems in healthcare domains.

#### 4.3.6. Rule-Based Methods

Rule-based reasoning (RBR) is a simple method to recognize patterns, anomaly and specific events based on the predefined and stored rules and conditions. Due to the basic task of the healthcare monitoring which is to find the obvious problem in physiological data, the RBR phase is necessary to apply on any wearable sensor framework [82]. A domain-specific expert system is a common rule-based method that defines and applies the expert conditions during data analysis [83–85]. A rule-based method is designed by [16] to detect different types of arrhythmias using ECG sensor data. In this work, a set of threshold-based rules is provided to apply to the ECG features in order to distinguish different arrhythmias such as Bradycardia, Tachycardia, Premature Ventricular Contraction (PVC), Premature Atrial Contraction (PAC) and Sleep Apnea. Obviously, the definitions of the conditions (range of values) for various features in ECG signal are acquired from expert system. Dealing with the general anesthetic problem is the subject of another research [86] which it has an online query processing algorithm which supports data stream queries asked by the expert of the system. The detection of the trends such as up, down, and flat in the time series data is based on the simple logical rules. The aim of this work is to directly monitor various requested trends by clinicians.

Since rule-based methods are using a set of provided rules by experts or the domain knowledge, they are able to perform anomaly detection task and to deal with unlabeled data sets to find unusual patterns. However, these methods are biased to the predefined rules in order to learn the features and behavior of the data itself. The rule-based methods are not adequate for decision making and prediction tasks as well as the personalized system with context aware targets. Moreover, they could not handle complex physiological data in real world experiments with unexpected patterns.

#### 4.3.7. Statistical Tools

Aside from the above mentioned techniques, other general statistical parameters and probability functions have been used for data mining techniques in the literature [39,53]. In the most of research in health monitoring systems, dealing with the simple statistical parameters (e.g., mean, variance *etc.*) or statistical functions e.g., risk function [61], factor analysis [68]) would be adequate to formulate the data and retrieve the expected information [38]. This kind of data analysis more or less applied on multi sensor networks in order to simplify the data features for model construction [30]. Commonly, statistical tools are modeling the input physiological data based on the statistical features and behaviors of health parameters. Thus, these models are able to detect the types of anomalies which are not easily seen by the expert. Nevertheless, domain knowledge and context metadata cannot be integrated with the statistical models. Despite that these tools are not reliable for decision making tasks, they have been used in clinical conditions with short term data in order to monitor the principal changes in the data stream.

#### 4.3.8. Frequency Domain/Wavelet Analysis

Analysis of time series data in the frequency domain enables to extract unseen patterns and trends across data [87]. Same as statistical tools, focusing on frequency domain, especially for wearable sensor time series data raises up meaningful information of data features. Both Fourier and wavelet transform analyses have been addressed in this literature mostly for feature selection step [29,52] and other tasks like estimation vital signs [88] and time series filtering and noise reduction in physiological data [54,89]. Frequency domain analysis is able to model and find complex patterns and detect the non obvious anomalies in the data. However, since the computational times in these techniques are high, it is not an appropriate to use these tools for real-time decision making systems. Furthermore, these models have not commonly applied for predication task due to the lack of domain knowledge in their models.

#### 4.3.9. Other Methods

Out of considered methods, there are other data mining techniques, which are roughly used in physiological data analysis. Some other examples are mentioned here: Bayesian network for classifying stress level [26,39] or for predicting patient's state [72], Fuzzy state machine for estimating the health status to provide alerts [68], logistic regression models [42] and association rules [41] to predict stress level and hospitalization of hemodialysis patients, respectively.

**Figure 4.**
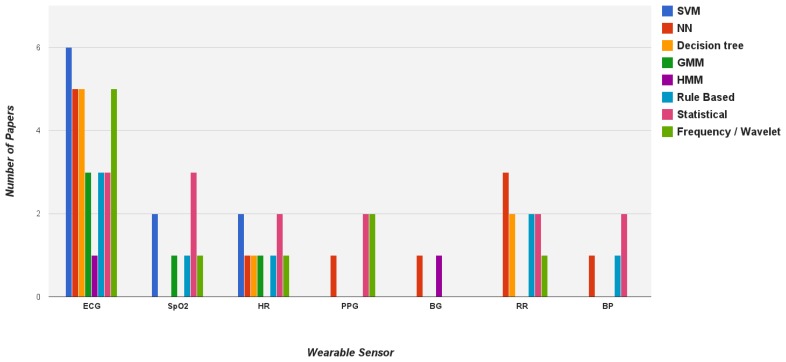
The outline of the application of data mining methods in relation to the vital signs measuring with wearable sensors.

According to the survey, Figure 4 presents the application of data mining methods in relation to the health parameters gathered with wearable sensors is depicted. It can be seen that ECG, HR, SpO_2_ and RR are the most complex health parameters where researchers have applied several data mining techniques to analyze these vital signs. Considering the data mining methods, SVM, NN, decision tree and frequency/wavelet are commonly used methods for wearable sensor data processing though they have seen more often only on the ECG health parameter.

## 5. Data Sets and their Properties

In any health monitoring system, having a robust data processing stage requires adequate information about the data itself. In other words, dealing with the type of input data and its properties is the prerequisite of any data processing system in order to handle the significant issues such as: selecting the proper data mining approach, designing and adjusting new method and features, and tuning the parameters of data analysis. To understand the main considered sensor data in the current research area, this paper examines the types of data and the method for gathering data that have been used in the experimental validation in the literature. This information gives the opportunity for the readers to distinguish the applied data processing methods based on the type of sensor data. This section provides detail on the type of data that has been collected using wearable sensors with considering two aspects: data acquisition approaches and data set properties.

### 5.1. Data Acquisition

Several input sources and data acquisition methods have been considered in the literature for wearable sensor data in health monitoring systems. Here, three major data gathering approaches have been identified such as experimental wearable sensor data, clinical or online databases of sensor data, and simulated sensor data. It is notable that depending on the mentioned tasks in Section 3, the data sources used in the literature were different.

- *Experimental wearable sensor data:* The papers which have developed the health monitoring systems have mostly used their own data gathering experiments to design, model and test the data analysis step [26,38,88]. In this case the gathered data are usually obtained based on the predefined scenarios due to the test and evaluate the performed results [36], but usually these studies do not provide the precise annotations and meaningful labels on physiological signals.
- *Clinical or online databases of sensor data:* Despite the attention of articles in this review is the role data mining on vital signs in health monitoring, several studies in this area have used the stored clinical data sets [31,89]. In other word, developed data mining methods is defined and designed for wearable health monitoring systems, but to evaluate quantitatively and test the performance of output decision of the framework, the most of the works used categorized and complex multivariate data sets with formal definitions and annotations by domain expert [30,40,45]. Very common example of online databases is PhysioNet [90,91] database which consists a wide range of physiological data sets with categorized and robust annotations for complex clinical signals. Several papers in the literature have used two main data sets in physioNet bank, MIMIC data sets (e.g., [53,60,61]) and MIT data sets (e.g., [27,54,92]) that contain the time series of patients vital signs obtained from hospital medical information systems.
- *Simulated sensor data:* For the sake of having a wide controlled analysis system, few works have designed and tested their data mining methods through shapely simulated physiological data [49]. Data simulation would be useful when the more focus of data processing method is on the efficiency and robustness of information extraction [28,57] rather than handling real-world data including the artifact, errors, conditions of data gathering environment, *etc.* Another reason to create and use simulated data is the lack of long term and large scale data sets [28] which helps the proposed data mining systems to deal with huge amount of data.

### 5.2. Data Properties

In addition to the data gathering methods, further properties of the wearable sensor data have been considered. In this subsection describes the investigated properties of data sets such as time horizon, scale, labeling, continuous/discrete, and single sensor/multi sensors data.

- *Time Horizon (long term/short term):* The length of time for considering data set measurements is a particular challenge for wearable sensor data in order to orientate the data mining techniques and the manner of data interpretation. Based on the purpose of any health monitoring system, the time horizon of data would be mattered. In this review paper, the time horizon of considered sensor data is categorized to short term and long term data. Some data analysis systems in healthcare were designed to process short signals such as few minutes of ECG data [74,92], a few hours of heart rate or oxygen saturation [60,61] and the measurement of blood pressures for a day [56]. On the other hand, dealing with long term data is the significant portion of some data mining methods for handling and processing lengthy period of sensor data. This period could be more than a number of days or a year of measurements. Blood glucose monitoring is an example of long term data analysis for the sake of right decision making [23,28].
- *Scale (large/small):* A big challenge of data analysis in any health monitoring system is the examination of the proposed method on more than an individual. Depending on the design of sensor network, data gathering, and the goal of decision making, the scale of subjects in the frameworks would differ. Here, the works considering a big number of subjects (patient or healthy) are counted as large scale studies [30]. The aim of these works is in addition to process the stream of data individually, they can handle the same data modelling for large scale of monitoring [31]. Due to the type of data mining tools, another group of papers focused on small scale of subjects (maybe one or at most 10 persons) in order to investigate the accuracy and correctness of results in proposed model [27,53].
- *Labeling (annotated/unlabeled):* Health monitoring systems need to evaluate their results in order to show the correctness of the decision making process. Due to have significant data analysis step, attention of the most research is given to annotated data. By considering the behavior of vital signs the domain expert would able to mark the data with several annotations such as arrhythmia disease [27], sleep discords [47], severity of health [61], stress levels [36], and abnormal pulse in ECG [32]. These annotations also acquired using another source of knowledge like electronic health record (EHR), coronary syndromes, and also history of vital signs [40]. Notice that these annotations are not necessarily marked on sensor data episodes, such some final decisions for the scenarios may be applied to the data set as labels [36,45]. On the other hand, working with unlabeled data leads to have unsupervised learning methods in order to extract unseen knowledge among raw sensor data. Some researches have been done on unlabeled data in order to consider uncontrolled situations for especially experimental data sets [39,44,88].
- *Continuous/Discrete:* Based on the design and architecture of wearable sensors the continuity of sensor data would be continuous or discrete. From the data mining point of view, the continuity of physiological data is important, since dealing with streams of sensor data has its own problems and challenges as well as analyzing sporadic measurements [30]. Due to the nature of vital signs, some of them are essentially counted as continuous such as ECG, heart rate and respiration rate [31,42] or discrete like blood glucose level [28]. Blood pressure data is an example which has been appeared in the literature in both continuous and discrete format [30,75].
- *Single Sensor/Multi Sensors:* Due to have robust decision making in any health monitoring system, the number of considered vital signs has a significant role to orientate and improve the results. Usually single sensor data have been used for specific analysis on individual physiological data such as ECG signal analysis [32,54,92] or blood glucose monitoring [23,28]. Besides the works focusing on physiological data mining gathered from a single sensor, most of the researches have used several sensors [30,40,44,67] to have global reasoning in health monitoring. Although using several wearable sensors in health monitoring frameworks are common, but few researches have really performed the multivariate data analysis in order to extract effective information through multi sensor data [30].

**Figure 5.**
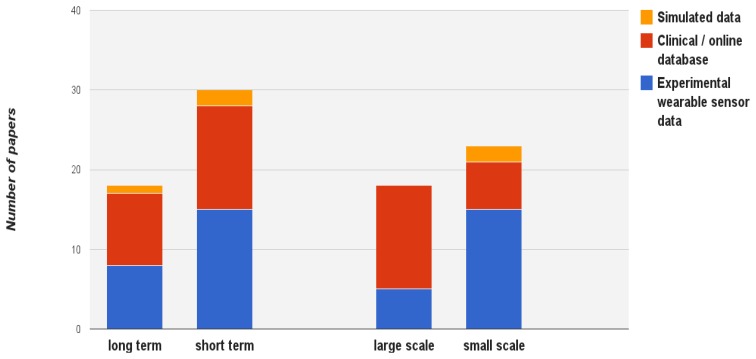
The distribution of works on two sensor data properties (time horizon and scale), with the relation to three types of data acquisition.

**Figure 6.**
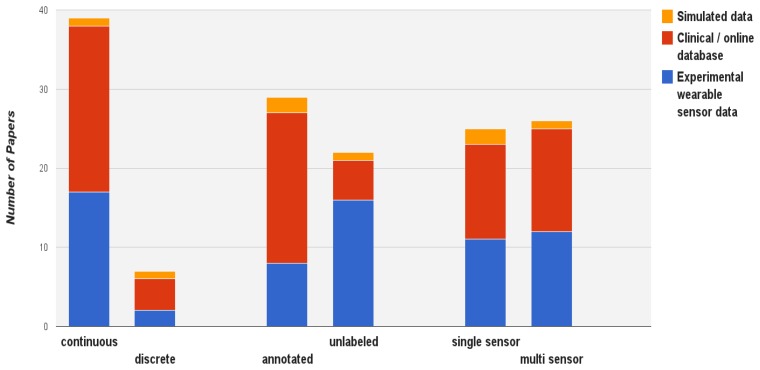
The distribution of works on three sensor data properties (continuous/discrete, labeling, single sensor/multi sensors), with the relation to three types of data acquisition.

A meaningful way to utilize the above elicited information about data sets is to consider their usage in the literature due to infer the role of data sources and data properties in data mining works. For this reason, the distribution of selected research for each data properties with the relation to the data set acquisition approaches have been shown in Figures 5 and 6. As can be seen from Figure 5, more attention has been given to short term data sets which can be easily recorded and analyzed for straightforward solutions in both experimental and clinical conditions. Beside, large scale data is typically more available in clinical settings and online databases where more resources are available.

Figure 6 depicts the distribution of works on the other data properties. In a clinical setting and online databases, data is mostly characterized as continuous and labelled (as access to the experts and other health profiles is readily available). Wearable sensor data collected in other contexts are rather unlabeled. In this field relatively little data is discrete and is usually collected in conjunction with other sensor modalities.

In Table 2, particular focus is placed on the relation between the vital signs and most of data properties in order to present the distribution of the current research based on the review articles. This table aims to help such researchers who are interested to find out some developed data processing approaches on the specific health parameters with characteristic properties of wearable sensor data. On considering time horizon, Since the short term data sets are easily acquired and processed, they have been more used than long term for the most of the health parameters excluding BG, BP which need naturally long term data to analyze. For the scale property, the large scale data sets are more popular than the small scale for most of the health parameters, except ECG and BG due to the fact that they contain the high frequency of available data sets in online databases. Most of the health parameters are used the annotated data sets rather than the unlabeled measurements excluding BG and RR, Since there is no adequate predefined annotations to be marked on specific temporal data. Most studies have been conducted on ECG sensor signal, where all types of data has been used *i.e.*, long/short term, large/small scale, and annotated/unlabeled data properties.

## 6. Discussion and Future Challenges

Data mining techniques have progressed significantly in the past few years and with the availability of large and open data sets, new possibilities for achieving suitable algorithms for wearable sensors exist. Still, despite these developments, their application to health monitoring is hindered by the challenges that are present in data from wearable sensors which create new challenges for the data mining field. This review paper has overviewed the data mining approaches used for wearable sensing devices that measure vital signs. For each approach, a reflection of its suitability for health monitoring was provided. From these reflections, the following guidelines for applying data mining methods was extracted:

- The selected data mining technique is highly dependent on the data mining task to be performed. According to the considered data mining tasks in Section 3, for anomaly detection task, SVM, HMM, statistical tools and frequency analysis are more commonly applied. Nevertheless, NN has not been addressed to detect anomalies. Prediction tasks on the other hand, have often used decision tree methods as well as other supervised techniques. It was shown that rule-based methods, GMM, and frequency analysis are not the most appropriate methods for predication due to the shortcoming in modeling the data behaviors. Finally, any decision making task needs a strong modeling and inferring system with a proper usage of the contextual information. So, the role of statistical methods in this task is less, but SVM, NN, and decision tree techniques have usually applied for the healthcare problems with decision making tasks with good success.
- The requirements for a real-time system should guide the selection of the data mining methods. To design a real-time health monitoring system, such methods like NN, GMM, and frequency analysis are not efficient for the sake of their computational complexities. But simple methods such as rule-based, decision tree and statistical techniques can quickly handle the online data processing requirements.
- The properties of the data set and experimental condition also influence the choice of method. Data mining methods (e.g rule-based, decision tree) have been used in clinical situation with controlled conditions and clear data sets, but the efficiency of them are not tested in real experiments of healthcare services. In contrast, some studies in the literature have used NN, HMM, and frequency techniques in order to handle complex physiological data and discover the unexpected patterns in real world situations.
- The level of supervision and labelled data is key factor to consider. Considering data properties in the selected research, such methods like SVM, NN, and GMM have been designed and justified to model long term data. However they could not deal with the unlabeled data in order to model in an unsupervised manner the raw data and features. For multivariate analysis of wearable sensor data, the methods working with domain knowledge such as rule-based, decision tree, and statistical tools are more usable, while GMM and HMM could not play this role in healthcare systems.

While these guidelines can assist those implementing technical systems to select appropriate methods for data analysis, the field is still challenged by a number of factors which have been discussed in this paper. These challenges are general challenges in the field of health monitoring and have emerged from the literature study performed here. They include:

- *Need for Large scale monitoring in non-clinical context:* One challenge is that still many applications using larger data sets and still consider monitoring in clinical contexts. This means that it will become of increasing importance for applications, which examine target groups such as elderly, healthy persons *etc.* to make significant effort in collecting reliable data sets for processing.
- *Dealing with annotated data sets:* Data mining approaches gain increasing attention in this field, open data sets as well as benchmark data sets become important in order to validate different approaches. Still however in this area few benchmark data sets are available. The last mentioned point raises a second challenge of how data annotation (labeling) can be best done for such target groups. The process of annotating data is expensive, time consuming and non-trivial considering long-term continuous data. To confront this challenge, an interesting avenue of study will be the efficacy of data mining in unsupervised contexts using unlabeled data sets. This applies both to the modeling as well as eventual preprocessing of data, where for example, unsupervised feature learning techniques [97] for time series data could show promise.
- *Multiple measurements:* Another challenge in this field is to exploit the multiple measurements of vital signs simultaneously. In particular, sensor fusion techniques which are able to consider dependencies and correlations between different vital sign parameters could assist in performing the main data mining tasks of prediction, decision making and anomaly detection. Some attention to this issue has been given in the literature such as [30].
- *Contextual information:* As hardware architectures allow for more parameters to be collected, more information could readily be collected. In addition, usage of contextual information to assist in data mining is of ever increasing importance. Such contextual information could include meta information about subjects such as weight, height, age, sex, history of vital signs, as well as history of previous decisions. It is also possible to automate the retrieval of high level information via available ontologies [98,99] and link this information to the data.
- *Reliability, level of trust to the system:* A caveat with automated data mining and black box learning methods is the amount of trust between the data analysis system and the experts who use the system for decision making tasks. For this reason, different approaches such as case based reasoning [100] may increase the level of trust by being explicit and referring to previous cases. However, it is still a big challenge that experts using healthcare services are not sufficiently interact with the reactive and proactive decisions made by the developed system.
- *Discovering of unseen features:* Still, important features of the data which may be unintuitive e.g., frequency domain features may be needed for providing proper analysis and uncovering important characteristics from the data which cannot be obtained by hand-engineered features. It also worth noting that in real world system such as home monitoring, it would be difficult to model the unexpected features with straightforward techniques.
- *Post-processing and Representation:* As a result of healthcare systems, an upcoming approach could use classical data mining techniques together with methods such as natural language generation which uncover trends in the data but also explain the process to both expert and non-expert users. Works such as [101,102] have demonstrated the possible uses of such systems in both clinical and experimental contexts.

In sum, the next few years present a subset of new and interesting challenges for the data mining and wearable sensors communities. As such systems are more readily deployed in real environments, experimental validation will need to consider realistic and long-term situations. Furthermore, specific challenges brought forth by the use of wearable sensors will also need to consider data mining methods that can address such challenges. The result is a move from applying out of the box data mining algorithms to a more concentrated effort towards novel data mining methods for the wearable sensors in health monitoring.

## 7. Conclusions

The aim of this study was to provide an overview of recent data mining techniques applied to wearable sensor data in the healthcare domain. This article has attempted to clarify how certain data mining methods have been applied in the literature. It also has revealed trends in the selection of the data processing methods in order to monitor health parameters such as ECG, RR, HR, BP and BG. Finally, particular attention was given to elicit the current challenges of using data processing approaches in the health monitoring systems. For this reason, this paper surveys several solutions provided by healthcare services by considering distinguished aspects. Namely this paper includes (1) data mining tasks for wearable sensors (2) data mining approach and (3) data sets and their properties. In particular, the review outlined the more common data mining tasks that have been applied such as anomaly detection, prediction and decision making when considering in particular continuous time series measurements. Moreover, further details of the suitability of particular data mining methods used to process the wearable sensor data such as SVM, NN and RBR has been described. Further study in this review paper focused on the sensors data sets features and properties such as time horizon, scale and labeling. Finally, the paper addressed future challenges of data mining while analyzing the wearable sensors in healthcare.

## Figures and Tables

**Table 1.** The summarisation of the most commonly used features of each wearable sensor data in the literature.

	**Time Domain**	**Spectral Domain**	**Other Features**
**ECG**	Mean R-R, Std R-R, Mean HR,Std HR [39], Number of R-Rinterval [27], Mean R-R, StdR-R interval [64].	Spectral energy [27,62], Powerspectral density [32], Low-passfilter [45], Low/highfrequency [39,64].	-
**SpO_2_**	Mean, zero crossing counts,entropy [48], Mean, Slope [61],Self-similarity [60].	Energy, Low frequency [60].	Drift from normality range [61], Entropy [60].
**HR**	Mean, Slope [61], Mean, Self-similarity, Std [60].	Energy, Low/high frequency [60],Low/high frequency [36],Wavelet coefficients of datasegments [45], Low/highfrequency, Power spectraldensity [42].	Drift from normality range [61], Entropy, Co-occurrence coefficients [60].
**PPG**	Rise Times, Max, Min, Mean [36].	Low/high frequency [36].	-
**BP**	Mean, Slope [61].	-	Rule based features [56].
**RR**	Mean, Min, Max [64].	-	Residual and tidal volume [64].
**Other**	Zero crossings count, Peak value, Rise time (EMG) [68], Mean, Duration (GSR) [36],Pick value, Min, Max(SCR) [51], Total magnitude,Duration (GSR) [39].	Spectral energy (EEG) [27],Median and mean Frequency,Spectral energy (EMG) [68],Energy (GSR) [36].	Bandwidth, Peaks count (GSR) [36].

**Table 2.** The outline of the categorization of selected papers based on the type of wearable sensor data and data set properties.

	**Horizon**		**Scale**		**labelling**	
	**long term short term**		**large scale small scale**		**annotated unlabelled**	
**ECG**	[31,35,47,48, 67,93]	[27,30,32,34,42,45,49,54,57,64,81,92,94]	[30–32,45,47, 48,67,74]	[27,34,39,42,44,49,52,81,94]	[27,32,45,47–19,52,54,57,81,92,94,95]	[30,34,35,39,42,44,46,62,93]
**SpO_2_**	[31,33,40,48, 93]	[44,53,60,61]	[31,40,48,60, 61]	[33,44,53]	[31,33,40,48,53, 61]	[44,60,86,93]
**HR**	[31,40,47,75]	[26,38,44,60, 61,96]	[26,40,47,60, 61,75]	[38,44,96]	[26,38,40,47,61, 75]	[44,60,86,96]
**PPG**	[67,72,93]	[36,53,88]	[36,67,72]	[53,88]	[36,53,67,72]	[88,93]
**BG**	[23,28,40]	-	[40]	[23,28]	[40]	[23,28]
**RR**	[40,67,75,86]	[30,34,42,46, 52,64]	[30,40,67,75, 86]	[34,42,46,52, 64]	[40,52,67,75]	[30,34,42,46, 64,86]
**BP**	[40,56,75]	[30,61]	[30,40,61,75]	[56]	[40,61,75]	[30,56]
**Other**	[36,51,55,68]	[33,41]	[33,51,55,68]	[36,41]	[51,55,68]	[33,36,41]
